# The journey within: mental navigation as a novel framework for understanding psychotherapeutic transformation

**DOI:** 10.1186/s12888-024-05522-8

**Published:** 2024-02-01

**Authors:** Mykyta Kabrel, Kadi Tulver, Jaan Aru

**Affiliations:** 1 Institute of Philosophy and Semiotics, University of Tartu, Tartu, Estonia; 2 Institute of Computer Science, University of Tartu, Tartu, Estonia

**Keywords:** Psychotherapy, Mental navigation, Self-analysis, Cognitive map, Metaphor, Spatial language

## Abstract

**Background:**

Despite the demonstrated efficacy of psychotherapy, the precise mechanisms that drive therapeutic transformations have posed a challenge and still remain unresolved. Here, we suggest a potential solution to this problem by introducing a framework based on the concept of mental navigation. It refers to our ability to navigate our cognitive space of thoughts, ideas, concepts, and memories, similar to how we navigate physical space. We start by analyzing the neural, cognitive, and experiential constituents intrinsic to mental navigation. Subsequently, we posit that the metaphoric spatial language we employ to articulate introspective experiences (e.g., “unexplored territory” or “going in circles”) serves as a robust marker of mental navigation.

**Methods:**

Using large text corpora, we compared the utilization of spatial language between transcripts of psychotherapy sessions (≈ 12 M. words), casual everyday conversations (≈ 12 M. words), and fictional dialogues in movies (≈ 14 M. words). We also examined 110 psychotherapy transcripts qualitatively to discern patterns and dynamics associated with mental navigation.

**Results:**

We found a notable increase in the utilization of spatial metaphors during psychotherapy compared to casual everyday dialogues (*U* = 192.0, *p* = .001, *d* = 0.549) and fictional conversations (*U* = 211, *p* < .001, *d* = 0.792). In turn, analyzing the usage of non-spatial metaphors, we did not find significant differences between the three datasets (*H* = 0.682, *p* = 0.710). The qualitative analysis highlighted specific examples of mental navigation at play.

**Conclusion:**

Mental navigation might underlie the psychotherapy process and serve as a robust framework for understanding the transformative changes it brings about.

**Supplementary Information:**

The online version contains supplementary material available at 10.1186/s12888-024-05522-8.

*“The only journey is the journey within.”*

- Rainer Maria Rilke.

## Background

Rooted in ancient civilizations [1] and shaped by the insights of pioneering psychologists [2–6], psychotherapy has emerged as a multifaceted approach to healing the mind and fostering personal growth. Over the last decades, there has been a notable increase in the number of individuals seeking psychotherapy as a means to address their psychological problems (e.g., [7]). This surge in popularity has transformed psychotherapy into a mainstream service, resulting in the emergence of new therapeutic approaches on a regular basis (for review, see [8]). Though extensive research has demonstrated that evidence-based psychotherapies are effective and cost-efficient for a wide range of psychological conditions [9–15], there remain several unresolved questions and ongoing debates.

One crucial area of contention revolves around understanding the specific mechanisms that contribute to effective treatment. While therapeutic interventions have shown associated changes in the brain [16–19], the precise neural pathways and mechanisms involved in bringing about therapeutic transformation are still under investigation. As Kazdin aptly stated, “After decades of psychotherapy research and thousands of studies, there is no evidence-based explanation of how or why even the most well-studied interventions produce change, that is, the mechanisms through which treatments operate.” [20] , p. 418. These complexities emphasize the dynamic nature of therapeutic practice and the need for a comprehensive framework for understanding and explaining the underlying mechanisms of psychotherapy. Here, we present such a framework, encompassing an explanation and refinement of both the applied psychotherapeutic methods and the resulting changes in neural mechanisms. The argument put forth is that certain mental processes during psychotherapy, specifically cognitive search or self-analysis, can be conceptualized as mental navigation.

### Mental navigation

Mental navigation is a nascent and rapidly developing concept that has emerged from the traditional study of spatial navigation in cognitive science [21–23]. Mental navigation encompasses our capacity to map and navigate through our thoughts, ideas, concepts, memories, and images, much like the way we navigate physical space [23]. Interestingly, over a century ago, William James drew a parallel between searching for forgotten ideas in memory and rummaging for lost objects in our physical surroundings [24] , p. 654. Indeed, throughout human history, there has been an implicit assumption that the inner search resembles the search in the environment as reflected in such ancient concepts as “Method of Loci” or “Memory’s theater.” In addition, this comparison is anecdotally supported by the fact that often spatial and conceptual knowledge are characterized as maps (e.g., [25, 26]) or networks (e.g., [27]).

Recent research provides empirical evidence supporting the validity of folk intuitions. For example, it was found that the search process within semantic memory demonstrates similarities to the exploration of physical space, characterized by a dynamic cognitive procedure that entails a balance between local exploitation and global exploration of information clusters [28, 29]. This parallel can be observed in the foraging behavior of animals as they navigate their environment in search of food patches [28]. Additional evidence supporting the existence of common cognitive processes includes shared neural correlates in search tasks [30], similar computational mechanisms [31], and the ability to prime search transitions from spatial to semantic domains [32], (however, see [33]).

The similarities between spatial and cognitive search extend beyond top-down cognitive processes and encompass bottom-up neuroscientific mechanisms as well. “Place cells” have been found in the hippocampus [34, 35], while “grid cells” have been revealed in the medial entorhinal cortex [36–38]. This research has demonstrated that place cells exhibit selective firing when an animal occupies a specific location within an environment, while grid cells form a hexagonal grid that covers the space. This phenomenon allows us, for example, to infer an animal’s current location by analyzing the firing patterns of these specialized cells. For instance, if a rat occupies one place, corresponding place cells will fire, and as it moves to a different place, different place cells will activate.

More recently, research has expanded our understanding of the involvement of place and grid cells in nonspatial tasks, challenging the traditional view of the hippocampus as solely responsible for spatial processing. Place cells have demonstrated their ability to encode task-relevant features of abstract concepts such as time, sound, odor, taste, and learned knowledge [39–43]. Similarly, the grid cell system, as evidenced by multiple studies, has been found to play a role in encoding visual categories, odor recognition, social hierarchies, word meaning, statistical regularities of events, the structure of complex narratives, concepts in abstract spaces, and semantic relations [37, 44–55].

The framework proposed by Buzsáki and Moser [56] offers an explanation for these findings, suggesting that cognition is an exaptation of physical action, meaning that with the incremental development of cognitive functions, the brain processes supporting our abilities to move through space become reused or “recycled” for other purposes, i.e., mental navigation of abstract representations. This adaptive process allows us to employ the same cognitive skills traditionally associated with spatial navigation to disengage from the physical world and navigate our mental landscape offline [57]. In brief, having internalized the model of the world, we can navigate it without physical movement.

### Spatialization of mental representations

Having introduced our capacity for mental navigation, it is essential to understand what constitutes a conceptual space that can be navigated. Despite the difficulties with precise cognitive space modeling, several prominent frameworks successfully tackle this challenge. One of these frameworks argues that our semantic space likely relies on high-dimensional geometries that represent multiple perceptual, functional, and abstract features. Thus, complex abstract concepts can be modeled using these high-dimensional spaces [58, 59]. For example, social space – akin to physical space – involves multiple continuous dimensions, like hierarchy and dominance (top and down), intimacy and familiarity (close and far), that serve as indicators of “social distance.” This conceptual modeling approach is also applicable to therapy. For instance, memories can be organized within low-dimensional spaces. Accessible or recent memories may be likened to those at the surface, whereas older or traumatic memories could be perceived as deeper and less readily accessible. Furthermore, there may be additional high-dimensional aspects within a specific memory, such as the emotional component, vividness, topic, etc.

The framework of semantic networks partially resembles the conceptual spaces theory as it allows for quantifying distance between categories and objects, both real and abstract (for a recent review, see [60]). By analyzing large text corpora, it is possible to quantify how close or far different objects are to each other in terms of meaning and frequency of occurrence together. For example, a cat and dog would probably be closer than a cat and elephant, which intuitively seems to reflect how the category of animals is organized in our minds.

Beyond attempts to model conceptual or semantic spaces, the grounded cognition framework deserves acknowledgment for its compelling argument that concepts cannot exist in an amodal vacuum; rather, they are intricately tied to our sensory experiences [61]. Even deeply abstract concepts, such as mathematical equations, are posited to be grounded in specific sensory encounters. This is exemplified in the mental representation of numbers through implicit associations with space, as seen in the manifestation of mental number lines, for example, on kindergarten walls or laptop keyboards [62]. Moreover, in early education settings (and sometimes later in life), the teaching of mathematics often involves spatial relations and real objects, e.g., “You have two apples and receive three apples – how many apples do you have?” This reliance on spatial representation, often involving manipulations with physical entities in space, underscores the fundamental role of space in our perception, learning, and reasoning [63, 64].

Alternatively, embodied cognition, alongside its extended version known as 4E cognition (embodied, embedded, extended, enacted), emphasizes our embodied and situated nature [65–67]. Rather than focusing solely on the brain, attention is directed to bodily experiences and interactions with the environment, primarily through physical actions. Thus, Lakoff and Johnson [68] introduced the notion of conceptual metaphor. It refers to a cognitive phenomenon where people understand and structure abstract concepts in terms of more concrete, sensorimotor experiences. It involves mapping elements of one conceptual domain (source domain) onto another (target domain), enabling us to understand and reason about abstract concepts in terms of more concrete and familiar experiences. Note that while spatial metaphors will be discussed in detail below, here we aim to emphasize the spatial nature of thinking beyond just linguistic expressions.

Specifically, Casasanto and Boroditsky [69] propose distinguishing between linguistic and mental metaphors. Linguistic metaphors involve expressions like “on the top of the world,” where spatial terms are used in language. On the other hand, mental metaphors involve the internalized, cognitive organization of abstract concepts in spatial terms without necessarily expressing them with spatial language. A concrete illustration of mental metaphors is our spatial arrangement of temporal concepts like “yesterday,” “today,” and “tomorrow” in left-right space [70]. This reveals a natural inclination to mentally organize abstract temporal concepts in a spatial manner, even if we do not explicitly use spatial metaphors when expressing them in language. Though it can be argued that spatial metaphors might not be overtly common, this distinction highlights how we consistently utilize spatial thinking, often without explicit awareness. In essence, physical space serves as a fundamental source domain for thinking [69–71].

Summarizing the last two sections, we argued that humans organize their abstract concepts through spatial representations, substantiated by various theoretical frameworks (e.g., conceptual spaces) [58, 59] and empirical evidence (e.g., mental number lines) [71]. Cognitive neuroscience studies on navigation further underscore the utilization of overlapping brain processes for structuring both physical and abstract entities with spatial organization [44–55]. The key proposition is that, besides having spatial maps for the physical environment, we possess conceptual maps representing abstract knowledge. This cognitive ability enables us to disengage from the physical world and mentally navigate through representational contents [56, 57].

Analogous to how we physically navigate from place to place finding our way around, we navigate mentally from one conceptual representation to another in order to reach more abstract solutions. Within the framework presented in this paper, this implies that individuals have the capacity to explore, expand, and reshape their internal cognitive landscape, mirroring the processes used for the exploration of physical environments. Given that during psychotherapy, clients actively participate in such a process, delving into their inner world, exploring uncharted territories, and making new connections, we hypothesize that clients engage in mental navigation within their own minds.

### Application of mental navigation in psychotherapy

**Fig. 1:**
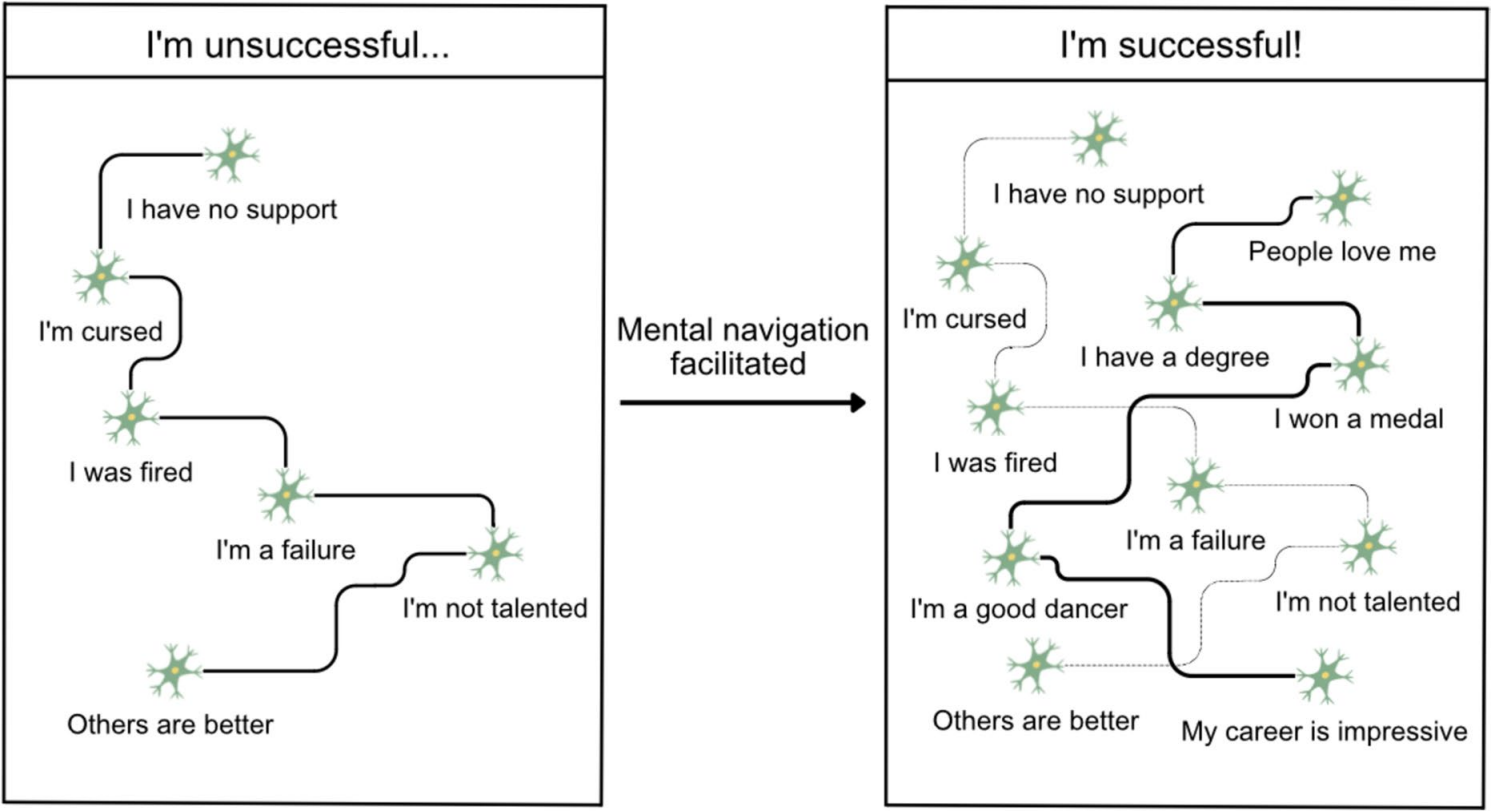
As depicted in the left picture, a preconfigured, rigid cognitive structure reinforces the belief “I’m unsuccessful.” However, by facilitating mental navigation, individuals can explore alternative concepts and establish robust connections that cause a transformation in the conviction while disengaging from the prior unproductive belief

Conceptual maps have emerged as valuable tools for navigating our cognitive space [21–23, 72]. However, similar to how a physical map can become outdated or damaged, leading us astray, our cognitive landscape can also exhibit rigid structures [73], dysfunctional connections, or maladaptive “shortcuts.” These problematic structures may manifest as automatic thoughts, fixed habits, emotional reactions, distorted self-perception, defensive or avoidant behavior, etc. Mental navigation, supervised and facilitated by a therapist, could allow one to identify and characterize these problematic junctions and, most importantly, enable one to update or restructure them efficiently. In the same way as cells in the hippocampal-entorhinal system can adapt and reorganize themselves when the environment changes [74–76], individuals – sometimes with the help of a psychotherapist – have the ability to restructure the connections between specific memories, concepts, or ideas. As a result, new perspectives emerge at the cognitive level (shown in Fig. 1).

In the following, we argue that in various scenarios, mental navigation might underlie the process of psychotherapy, thus providing a useful framework for therapists to rely on. For instance, viewing the work with ruminative thoughts or inflated cognitive biases through the lens of mental navigation can provide a valuable perspective. For example, individuals experiencing depression or anxiety can experience ruminative thought patterns and a feeling of “going around in circles.” They may be “stuck” in maladaptive pathways and network connections, basically having fixed an automatic shortcut that needs to be changed. This change can be facilitated by exploring and establishing new, more effective connections using various techniques, for example, “cognitive restructuring” in Cognitive-Behavioral Therapy (CBT) [77].

Another example encompasses the so-called “jumping to conclusions” (JTC), a phenomenon wherein people reach unwarranted conclusions with limited information. There are several papers discussing the role of JTC in the development of psychosis (e.g., [78–80]) or schizophrenia (e.g., [81]). Additionally, JTC contributes to impaired emotional recognition and the tendency to make internal attributions for negative events [82]. This implies that clients, guided by an underlying maladaptive belief system, often come to negative conclusions without an attempt to search for a more constructive path. One of the options to bridge the gap between realistic conditions and clients’ convictions is the “disputing irrational beliefs” technique in Rational Emotive Behavior Therapy (REBT) [83], which helps one to come to adequate conclusions by searching for a different path within a broader “map.” In short, such approaches as CBT or REBT may utilize mental navigation to focus directly on identifying and making explicit cognitive structures that were acquired implicitly and negatively influenced one’s thinking. Through the process of modification, new and effective shortcuts could be established, being readily available when a new situation comes [84, 85].

Furthermore, utilizing the conceptualization of mental navigation in psychotherapeutic methods extends beyond just the cognitive-behavioral tradition. For instance, the free associations technique [86, 87] that is employed in the psychodynamic tradition can also be viewed from this perspective. In certain instances where it may be difficult to openly discuss thoughts and memories of distressing events, the free exploration of one’s stream of thoughts and associations can allow individuals to navigate their psychological landscape without the constraints of a predetermined structure. By doing so, this method facilitates the emergence of sudden and unexpected connections that were previously unimaginable, resembling the phenomenon of insight or the “Aha” moment [23, 88]. This process allows old pathways to be recognized and severed, paving the way for the reconstruction and emergence of a new and more cohesive understanding or narratives. It can be metaphorically compared to freely wandering in a familiar environment where one typically follows the same paths but now notices alternative routes that may be more effective or less energy-consuming.

### Frameworks compatible with mental navigation

Although using the mental navigation concept in the context of psychotherapy is novel, other hypotheses have been offered to explain some of the same mechanisms. For example, some links can be observed between the concept of mental navigation and the memory reconsolidation paradigm [89–92]. Specifically, Ecker & Bridges [91] argue that the existence of a client’s symptomatology “indicates the presence, outside of awareness, of distress-laden emotional learnings *(schemas, mental models)* … and the optimal process of psychotherapy consists of guiding the profound unlearning of the symptom-generating emotional learnings” [p. 298, our italics]. Thus, in the process of reactivating old memories, mental navigation could help one to substitute or establish new semantic structures, schemas, or mental models (i.e., conceptual maps in our paradigm) that lead to more adaptive ways of interpreting events and, in turn, more appropriate emotional responses [89]. In other words, mental navigation might help reconsolidate old memories with new information.

Additionally, in line with the mental navigation concept, the experiential perspective in e.g., emotion-focused therapy (EFT), views a core change mechanism in psychotherapy through more complete processing and awareness of emotion and through the transformation of emotion schemes [93–96]. Hence, throughout this process, mental navigation could aid one in becoming more consciously aware of their emotions by attending to bodily sensations, feelings, searching suitable words or images to symbolize them, and exploring what the emotion means.

Notably, the role of the therapist is a crucial aspect of the mental navigation process. For example, when recalling a traumatic memory, they can help the client attend to some contextual information that might not have been available for the client at the time of the trauma [97], thus incorporating more adaptive cognitive structures. Expressing and describing one’s thoughts, memories, or emotions during psychotherapy helps to create a more coherent narrative of what occurred, what the emotional and behavioral reactions were, and how they could be different [98]. Therapists, thereby, could play the role of “skilled travel companions” in this process, helping to navigate an intricate mental space of the traumatic experience. Drawing upon a different, perhaps broader perspective, therapists have the capacity to reinterpret situations and the client’s emotional responses, potentially leading to novel insights and perspectives [89].

Last but not least, mental navigation could play a role in another powerful framework applied to psychotherapy: predictive processing [99–101]. A number of recent papers have explored the potential implications of predictive processing for psychotherapy and mental well-being (e.g., [91, 102–105]). It is suggested that a discrepancy between the client’s preexisting mental schemas and newly identified ones during psychotherapy may cause a “prediction error” [88, 91], leading to reconstruction or updating of the system on different levels depending on the significance and depth of the revision [88]. Supporting this suggestion, several studies have indicated that a prediction error is crucial for a memory to become labile and modifiable [106–108].

We suggest that mental navigation might serve as a prerequisite for triggering prediction errors during psychotherapy. This process can be outlined as follows: an individual navigates their cognitive space, exploring and recognizing preexisting structures and schemas, consequently juxtaposing them with alternative ones. These fresh perspectives can be introduced by a therapist and – if done skillfully – could evoke prediction errors, leading to cognitive restructuring and a shift on the phenomenological level. However, note that the introduction of a novel perspective is not a pivotal factor. What remains essential, in our view, is the act of recognizing and becoming aware of inadequate functioning or a conflict in existing mental structures [88]. The advice of a therapist might not work until the discrepancy is revealed. Therefore, to evoke a shift, the initial step often involves engaging in mental navigation. In other words, the experience of prediction error does not *merely* entail encountering a new perspective but rather a genuine realization of the existing discrepancy.

As discussed, mental navigation might underlie the therapeutic work on multiple psychological conditions within several prominent frameworks, and there are undoubtedly many more beyond those mentioned. Irrespective of the specific approach and condition, the outcome of the techniques incorporating mental navigation entails restructuring, broadening perspective, gaining a deeper understanding, or even constructing a new narrative – all achieved through exploration. Hence, we contend that by viewing psychotherapy as a journey in the cognitive space, we can better grasp the shared requirement of navigating through representational contents. Just as any journey may encounter obstacles, junctures, impasses, and destinations, psychotherapy can be seen as a process that involves overcoming challenges and reaching a desired outcome. In this context, therapists, as already noted, can play the role of skilled travelers, providing support in effective navigation using therapeutic techniques as symbolic maps or compasses.

### Metaphor in psychotherapy

In the preceding sections, we intentionally emphasized spatial notions due to their frequent use by clients in the form of metaphors or abstract concepts during self-analysis, as supported by empirical evidence [109–119], see also the findings of this study and anecdotal observations. Metaphoric expressions are integral to human communication across diverse cultures and contexts. To illustrate their prevalence, the research on 47,000 words revealed that 18.5% of metaphors are employed in academic writing, 16.4% in news, 11.7% in fiction, and 7.7% in conversations [120].

In their pioneering work, Lakoff and Johnson [68] introduced the conceptual metaphor theory. As briefly noted above, conceptual metaphor refers to a phenomenon in which abstract concepts are understood and structured in terms of more concrete, sensorimotor experiences. It involves mapping elements of a source domain (e.g., space) onto a target domain (e.g., mood) as instantiated in phrases like “I’m feeling low.” This allows individuals to comprehend and reason about abstract concepts through the lens of more tangible experiences.

For many years, metaphoric language has also been considered important for the processes and outcomes of psychotherapy (for a recent review, see [121]). Indeed, it could be argued that the primary tool in therapy that helps achieve its outcome is language. Metaphoric expressions can thus have many roles during psychotherapy, and the intentional and skillful utilization of metaphors, as well as paying close attention to metaphors produced by clients, are often encouraged and promoted during the training of psychotherapists [121–123].

The research on metaphor, including metaphoric speech within psychotherapy, emphasizes several types of metaphors. Among the most important are conventional and novel types of metaphors. Conventional metaphor refers to metaphoric expressions that are widely established and frequently used in a given culture and linguistic community (e.g., “time is money”). These metaphors have become so ubiquitous that sometimes they may not strike the audience as figurative. On the other hand, novel metaphors are creative and inventive linguistic entities that are not widely used and are sometimes produced at the moment (e.g., “this classroom is a zoo”). In the context of therapy, the production of novel metaphors can be increased, especially in moments of high engagement and close self-reflection [116, 124]. This tendency may arise from the exploration and unveiling of novel information that has not been previously conceptualized in words. Consequently, figurative expressions are utilized, drawing on similarities between new experiences and familiar objects or situations.

A study by Hill and Regan [125] revealed that, in post-session evaluations, clients rated the metaphors employed by therapists as more helpful than methods that did not involve the use of metaphors. The effectiveness of metaphors in psychotherapy is particularly pronounced when imbued with personal meaning or employed for case conceptualization. This strategic use enables the disentanglement of complex conceptualizations into more digestible forms, fostering change by reshaping perspectives and enhancing memory retention of the metaphor [119, 126, 127]. The application of well-designed metaphors has been proposed as an effective means to alter distorted views held by clients, providing access to meaningful structures that might otherwise resist traditional approaches [128].

Another important role of metaphors in psychotherapy is the ability to facilitate affective expression [111, 124, 129–131]. Studies have demonstrated a correlation between the use of metaphors and emotional change, especially when expressing intense emotions [132, 133]. This process enhances understanding and provides individuals with the ability to manipulate or handle their emotions more effectively due to their actualization through metaphor [132, 134]. Furthermore, the use of metaphors addresses the challenge of expressing vague emotions precisely and offers a concise and broadly understandable manner to articulate complex and nuanced emotional experiences [134].

In psychotherapy, a significant emphasis is placed on the collaborative construction of metaphors between therapists and clients, a process known as co-construction [135–140]. Research suggests that the effectiveness of metaphors is heightened when both the client and therapist share a similar preference for metaphoric language [136]. Clients rated sessions with the use of metaphors as more helpful compared to other therapeutic approaches, particularly emphasizing moments when metaphors are created and developed collaboratively and iteratively [141]. The critical aspect of co-construction lies in how attentively the client engages with and further develops the metaphors introduced by the therapist and vice versa [121]. While co-construction alone does not guarantee positive outcomes, it underscores the importance of using metaphors in therapy with specific aims, ensuring that they hold meaningful significance for both parties involved [136].

Regarding the impact of metaphor usage on therapy outcomes, there is no unanimous consensus or straightforward answer on whether employing metaphors leads to enhanced results [121]. A recent review concludes that while there is limited evidence supporting improved outcomes in both the short and long term [116, 142–145], the role of metaphors is notably evident in the immediate context of therapy sessions [121]. However, in some cases, central metaphors can be assimilated into the client’s thinking if they are particularly meaningful [121].

Of particular interest to this study are metaphors related to space or movement [146, 147]. One essential function of movement metaphors is thought to be realized when a physical movement is used as a source domain to depict ongoing processes in therapy, including progress or absence thereof. Given the temporal and goal-oriented nature of therapy sessions, one of the prevalent and expected metaphors is “therapy is a journey” [148]. This concept encapsulates the idea that the therapeutic process resembles a voyage with identifiable stages, challenges, and progression. For example, Sarpavaara and Koski-Jännes [143] conducted a study examining the spontaneous use of metaphors by individuals undergoing substance abuse treatment. Their findings indicated a positive correlation between the use of the “journey” metaphor and favorable treatment outcomes.

Furthermore, frequent references to past or future situations evoke the metaphors related to directions, e.g., forward (“put the foot forward”), backward (“make a step back”), upward (“get up there”), downward (“spiraling down”), cycle (“go in circles”), uncertainty (“wandering”), and containment (“dig in”), among others [149]. Interestingly, the study by Tay [149] revealed that during the discussion of a client’s issues, the metaphors related to “sideways” and “uncertain” directions are used more frequently than “forward” directions. In addition, as psychotherapy progressed toward its conclusion, clients exhibited an increased usage of metaphors linked to personal development (how they “grew”), travel (how they “moved”), and organizational processes such as cleaning (how they “sorted things out”) [150]. One notable implication of this study is that, after successful therapy, clients internalize these metaphors, fostering a sense of confidence. This equips clients with metaphorical frameworks that mirror the transformative “path” they navigated throughout their therapeutic journey.

Notably, despite the extensive range of theoretical and empirical studies on metaphors within the field of psychotherapy, there are only a few studies that exclusively focus on spatial metaphors [146–150]. Having highlighted the significance of mental navigation in therapy, we believe that it is reasonable to concentrate more specifically on spatial metaphors and explore their unique implications and applications within the therapeutic context.

### Present study

As outlined in the previous sections, a significant part of our cognition exhibits a spatial nature. Substantial evidence supports the notion that even abstract concepts display spatial organization, with underlying neurocognitive mechanisms facilitating mental navigation akin to spatial exploration.

By integrating insights from conceptual/semantic spaces [58, 59] and grounded/embodied cognition frameworks [65–67], along with neuroscientific evidence highlighting the encoding capabilities of place and grid cells for abstract concepts in spatial codes [44–55], it is reasonable to propose that individuals, during therapy, leverage their navigational abilities to explore and reshape problematic mental structures within the conceptual spaces. This proposition offers an explanation for why these therapeutic explorations often feel and are metaphorically conceptualized as journeys, with spatial metaphors becoming an integral part of the therapeutic process.

Specifically, we hypothesize that spatial (both mental and linguistic) metaphors play a crucial role in therapy, serving as facilitators of the self-analysis process. The hypothesis posits that during tasks involving close self-examination (and other intellectually or metacognitively demanding activities), in addition to the phenomenology of the experience resembling physical navigation, there is an increase in explicit spatial language use and implicit spatial conceptualizations. This heightened usage of spatial language might arise from the necessity for mental navigation within spatially organized concepts, likely influenced by neurocognitive mechanisms associated with physical space. The specific suggestion is that increased spatial language usage acts as a tool for describing or expressing the mental navigation process and serves as a strong indicator of mental navigation with distinct phenomenology and associated brain mechanisms, potentially serving as a proxy for estimating when a cognitive shift has occurred.

In order to test this hypothesis, we conducted research using both qualitative and quantitative analysis. For the qualitative aspect, we examined a database of psychotherapy transcripts, focusing on the occurrence of metaphors related to navigation during self-analysis, such as “being at a crossroads” or “going in circles.” For the quantitative part, we compared the usage of this navigational language between psychotherapy sessions, more casual conversations, and scripted artistic conversations.

In the subsequent sections, we will describe the methodology employed to analyze the database, including the identification and categorization of navigation-related concepts. Then, we will present our findings, discussing the prevalence and patterns of these metaphors across the transcripts. Additionally, we will explore the implications of these concepts for understanding the cognitive processes involved in psychotherapeutic transformation. Finally, we will discuss the implications of our findings for the field of psychotherapy, highlighting the potential benefits of explicitly addressing and leveraging mental navigation in therapeutic interventions. We will also address the limitations of our study and propose avenues for future research to further elucidate the mechanisms and effectiveness of mental navigation in psychotherapy.

## Methods

### Qualitative methods

We accessed the database of psychotherapy transcripts furnished by Alexander Street Publisher [151], which consists of 2111 separate transcripts in the English language. Each transcript contains a conversation between a psychotherapist and a client, which was audio recorded and then transcribed into text. The content of a transcript typically looks as follows [152]:

“CLIENT: So I want to tell you something that I've been keeping from you and I guess was from out of embarrassment or whatever.

THERAPIST: Sure.”

The transcripts encompass different mental states like depression, anxiety, BPD, OCD, ADHD, PTSD, sexual dysfunction, relationship issues, and many more. It also represents different psychotherapeutic approaches and modalities, including but not limited to psychoanalysis, CBT, REBT, person-centered therapy, humanistic psychotherapy, and mindfulness techniques.

To begin with, we downloaded all the transcripts as separate files, assigning each file a number from 1 to 2111. Then, we mixed the files so that the sequence became random. Consequently, we used a systematic random sampling approach, selecting every 19th file from the dataset until the desired sample size of 110 transcripts (approximately 5.22% of the entire volume) was achieved. This random sampling approach ensured that our findings could be generalized and encompass diverse therapeutic techniques, psychological conditions, and personalities. During the examination, the primary objective was to explore and uncover the ways in which therapists and clients describe their introspective experiences using spatial language.

To identify metaphors in the database, we relied on the Metaphor Identification Procedure (MIP) [153, 154], which encompasses several steps. First, we read the transcript to establish a general understanding of the meaning. Then, we collected potential metaphors by identifying words or expressions that suggest a departure from literal language. To accomplish this, we examined the surrounding context, encompassing the previous, current, and subsequent paragraphs around the potential metaphor, to understand whether the word was employed in its literal sense or as a substitution of direct meaning for figurative meaning. Consequently, we determined the source and target domains of each potential metaphor. Lastly, we identified expressions as metaphors if their source and target domains were in discrepancy, i.e., one phenomenon was used to describe another phenomenon from a completely different domain.

For instance, phrases like “this is an unexplored territory for me” were indicative of metaphoric language. In this case, the source domain is an unexplored physical territory, while the target domain is a feeling of uncertainty or absence of knowledge. In turn, we excluded instances where the language use was literal or irrelevant to mental navigation, such as “When we were hiking in the forest, we went in circles,” and retained only those cases that aligned with the framework of mental navigation, like “I want to find an answer, but I’m going in circles.”

Subsequently, we manually (CTRL+F) performed a keyword and key statement search across all 2111 transcripts (we merged separate files into one large “.txt” file) based on the identified metaphors and concepts. The analysis revealed substantial utilization of spatial language. In the results section, we will present selected illustrative examples and provide further interpretation to shed light on their importance.

### Quantitative methods

To quantitatively test the hypothesis, we utilized three databases, all in the English language: the aforementioned database of psychotherapy transcripts by Alexander Street Publisher, the Spotify Podcasts transcripts database [155, 156], and a substantial collection of movie subtitles [157] (see Table 1 for detailed information). The main objective was to determine if the use of spatial language to describe one’s experiences is more common in psychotherapy compared to other types of dialogical conversations. We selected the database containing podcast transcripts as it, in contrast to psychotherapy, represents non-formal everyday conversations covering various topics, from entertaining to informative, and are presented in diverse styles, from friendly to professional. The subtitles database was chosen to represent scripted fictional text, which may incorporate more figurative language due to its artistic context and goals.

**Table 1:** Databases information

Database	Quantity	Word count
**Psychotherapy**	2111 session	12,883,335
**Podcasts**	2061 episodes	12,456,750
**Subtitles**	Not defined	14,182,762

In terms of compositional focus, psychotherapy transcripts are primarily dedicated to personal topics, whereas the other two datasets encompass a broader spectrum of themes. It is reasonable to assume that all three datasets reflect a diverse representation of demographic and social backgrounds. Therapy clients range from unemployed individuals to doctoral students to business owners, illustrating a wide spectrum of socioeconomic status. Analogously, podcasts feature recordings from both amateur content creators from lower and middle-income classes as well as professional podcasters who represent the higher-income class. Lastly, movies encompass different genres, various contexts, and dialogues between individuals from different social classes, employing varying linguistic vocabularies.

In terms of temporal scope, the psychotherapy transcripts encompass a period from 1970 to 2015, providing a longitudinal perspective on language usage within therapeutic settings. Notably, the data is not explicitly labeled and, in most instances, provides the publication year rather than the recording date, precluding a precise determination of the proportion of sessions per year. The movie subtitle database, while not explicitly time-stamped, also encapsulates a broad temporal range reflective of their respective genres (from at least 1970 to the present, as observed during the qualitative analysis). In contrast, the podcast dataset comprises conversations recorded between January 2019 and March 2020. Therefore, the psychotherapy and subtitle datasets are well-suited for comparison in terms of timeframe. However, the limited temporal range of the podcast dataset, representing conversations from a relatively short period, constitutes a potential limitation when compared to the other two datasets. In regard to cultural background, all datasets represent conversational corpora of Western anglophone cultures exclusively.

We recognize the inherent challenges in identifying metaphors with precision, as a spectrum of ambiguity exists in their usage. For instance, metaphors can be broadly categorized into conventional and novel forms. Additionally, we encounter instances of metonymy (e.g., “The White House”) and idioms (e.g., “to bite the bullet”). This study did not intend to delve into the intricacies and distinctions between these linguistic phenomena but rather focused on identifying the presence and frequency of figurative spatial language usage per se. Accordingly, our unified aim was to determine linguistic expressions in which space was used in its figurative form, i.e., to describe something that is not spatial in nature, e.g., thoughts or feelings. In order to achieve this, as already mentioned, a close contextual analysis utilizing the Metaphor Identification Procedure (MIP) [153, 154] was performed for the identification of spatial language in each database.

Note that the data used in both qualitative and quantitative analyses consisted of transcripts provided by third parties or were publicly available. All transcripts were thoroughly anonymized prior to our obtaining the materials. Since we did not directly collect the data and it was already anonymized, we did not seek ethics approval or informed consent from our institution. We acknowledge that the third parties have followed their own ethical procedures during the data collection and anonymization process. In addition, we ensured ethical research practices by adhering to guidelines for data usage and citation.

To begin, we conducted a manual search (CTRL+F) in each of the databases (large “.txt” files) to calculate spatial/navigational concepts. These concepts were defined based on the qualitative analysis of psychotherapy transcripts as described earlier. Subsequently, we manually filtered out cases where the spatial language was directly used to describe physical space, retaining only instances where it was used metaphorically to describe self-analysis or introspective experiences. However, during manual categorization, we encountered challenges in distinguishing when a spatial metaphor was used introspectively versus figuratively or to describe others’ experiences. For instance, phrases like “to navigate relationships” used the word “navigate” metaphorically, but the introspective connotation was not clear. We were aware of these complexities, and to address potential biases, we performed another search with a different categorization method. In the second categorization, we included all cases of metaphoric use of spatial concepts, regardless of the state in which it was produced (i.e., during close self-examination or during regular pre- or post-session chatter). As a result, we obtained two sets of data: “Introspective spatial concepts,” which includes only those metaphors produced during close introspection, and “All spatial concepts,” which includes all instances of metaphorical word use throughout the session.

Next, we normalized the frequencies of each metaphor for each database in Python custom calculation using the formula: (number of metaphors per database / number of words per database) * 1000. This approach allowed us to calculate the frequency of metaphors per 1000 words in each database, facilitating objective comparisons.

An additional concern is that psychotherapy involves discussing abstract concepts or previously undefined experiences and feelings, which may lead to the use of more figurative language. Thus, it remains uncertain whether this heightened usage specifically pertains to spatial metaphors or encompasses metaphors in general. To address this question, we conducted a third analysis focused on identifying and comparing non-spatial metaphors in each database. To define non-spatial metaphors, we referred to previous research papers [109, 110, 121] and conducted a search using GPT-3.5 [158] with the query: “The most common everyday metaphors in English” (see Table 2 for lists of both types of metaphors). Following a procedure similar to the one used before, we excluded cases of direct metaphorical word use and normalized the frequencies of non-spatial metaphors per database. This analysis yielded a third set of data, referred to as “Non-spatial metaphors.”

**Table 2:** The spatial and non-spatial concepts categorized based on the qualitative analysis

Spatial/navigational concepts	Non-spatial/navigational concepts
My mind goes to…	“Butterflies”
To navigate	Puzzle
Feeling (dis)oriented	Burden
Maze	Piece of cake
Pathway	Open book
Going in circles	Broken record
Mapping, mapped	Cold feet
Exploration	Wake-up call
New, unexplored, unfamiliar, uncharted, unknown territory	Silver lining
Mental Space, headspace	Busy bee
Dead end, impasse	Mind’s eye
Crossroads	Pressure cooker
Bypass, detour(ing)	Emotional rollercoaster
Back of my mind	“Chemistry”
Dark place	“Struggling”

Note that the spatial component in each metaphor can be interpreted differently. We have categorized metaphors into two groups based on their utilization of space in expressing the process of self-analysis. The first group comprises metaphors directly involving or referencing space as a primary source domain. For instance, we include expressions like “going in circles” or a “maze” to the first group due to our observations based on the qualitative analysis of the transcripts in which people refer to mazes as conceptual places that are difficult to navigate, while they “go in circles” when they report that they come to the same thoughts each time. In contrast, the second group includes metaphors that, despite potentially using space indirectly, do not primarily rely on navigation to convey the process. For instance, terms like “chemistry” may denote the therapist-client connection without explicitly invoking or utilizing space, and the same principle applies to metaphors such as “struggling” (something requiring physical effort rather than navigation) or “piece of cake” (something primarily related to pleasure). While space may play a secondary role in these metaphors, it is not their primary source domain. Based on that, we identified metaphors to be used and separated them accordingly. The types of metaphors identified by us are shown in Table 2.

However, to support the impartiality of the analysis, we also conducted a survey among 10 subjects unfamiliar with this article. We provided them with a randomized list of metaphors and asked them to separate them into “metaphors associated with space or navigation” and “metaphors not associated with space or navigation.” In cases where a metaphor seemed ambiguous, participants were asked to label it as an “ambiguous metaphor” and exclude it from the previous two categories. We then identified metaphors that achieved a consensus of at least 80% agreement among participants for categorization. The results of this categorization are presented in Table 3. For convenience purposes, in the results section, we will refer to the results of the analysis based on the participants’ choice as “impartial categorization.”

**Table 3:** The impartial categorization of spatial and non-spatial metaphors by the participants of the survey

Spatial/navigational concepts	Non-spatial/navigational concepts
To navigate (100%)	“Butterflies” (80%)
Crossroads (90%)	Open book (80%)
Dead end, impasse (90%)	Pressure cooker (80%)
Maze (90%)	I’m (was) struggling (80)
Going in circles (100%)	Broken record (80%)
New, unknown, unexplored, uncharted, unfamiliar territory (80%)	Busy bee (90%)
Bypass, detour(ing) (90%)	Cold feet (80%)
Pathways (90%)	Wake-up call (80%)
My mind goes… (90%)	Piece of cake (80%)
Rock bottom (80%)	Silver lining (90%)
Mapping, mapped (80%)	Chemistry (90%)
Light at the end of the tunnel (90%)	

The percentages enclosed in parentheses represent the level of agreement among participants regarding whether a specific concept belongs to either of the two categories

Following the collection of three datasets, we conducted a statistical analysis. For each of the tests, we utilized Python “scipy.stats” libraries. Initially, we performed a Shapiro-Wilk test to determine the normality of the data. The test results indicated that the data in each dataset is not normally distributed. Hence, we utilized the Kruskal-Wallis test (using a significance level of 0.05), which is instrumental in comparing the medians of metaphor frequencies across datasets, ensuring the validity of our comparisons where data deviates from normality. As the Kruskal-Wallis test showed significant differences between the three groups, we conducted post hoc Mann-Whitney U tests (significance = 0.05) to determine the precise differences between datasets where relevant. Finally, we corrected the *p*-values using the Holm-Bonferroni correction to adjust the results for multiple comparisons.

## Results

### Quantitative analysis

#### Introspective spatial concepts

**Fig. 2:**
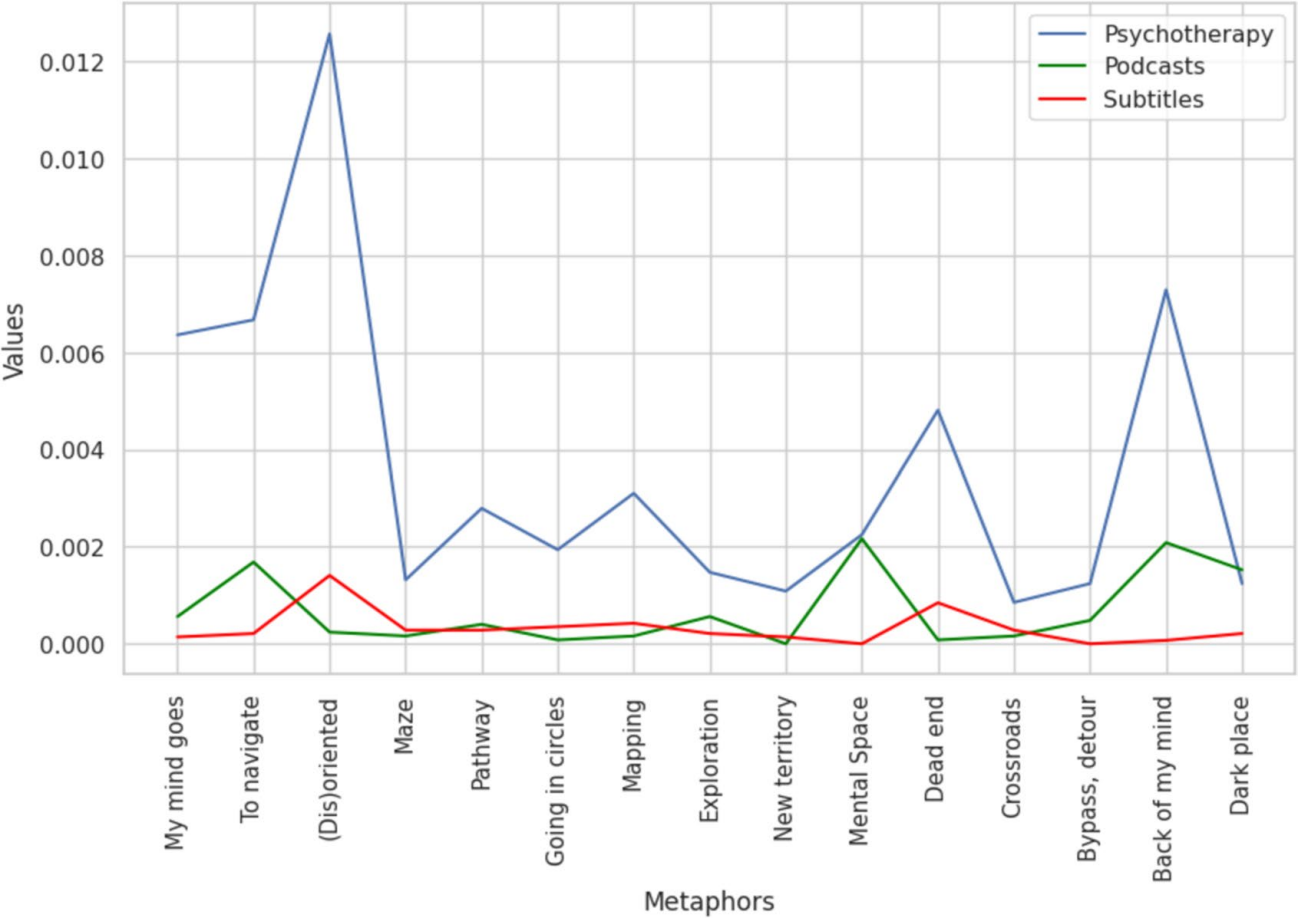
Frequency of occurrence of spatial/navigational concepts in introspective connotation between three datasets

The first dataset comprised normalized frequencies of navigational concepts used in introspective connotation during psychotherapy, podcast conversations, and movie conversations. The Kruskal-Wallis test revealed significant differences between the three datasets (*H* = 23.023, *p* = < .001). Mann-Whitney test showed further differences between psychotherapy and podcasts (*U* = 199.0, *p* < .001, *d* = 0.731), as well as between psychotherapy and movies (*U* = 220.0, *p* < .001, *d* = 0.911). On the contrary, there was no significant difference in navigational metaphor usage between podcasts and subtitles (*U* = 141, *p* = 0.244, *d* = 0.323) (shown in Fig. 2). The effect observed in psychotherapy transcripts was not driven by a few concepts. If we omitted the top three most frequent introspective spatial concepts from our analysis, they were still more prevalent (*H* = 18.289, *p* = < .001) in psychotherapy transcripts than in podcasts (*U* = 124.0, *p* = .005, *d* = 0.666) and movies (*U* = 144.0, *p* < .001, *d* = 1.033). As the objective categorization of metaphors on spatial and non-spatial posed challenges, we also conducted additional analysis on the “Impartial categorization.” This analysis did not affect the results, and we could still observe significant differences (*H* = 19.673, *p* = < .001) between psychotherapy and podcasts (*U* = 116, *p* < .001, *d* = 0.961) and between psychotherapy and subtitles (*U* = 121, *p* < .001, *d* = 1.067).

#### All spatial concepts

**Fig. 3:**
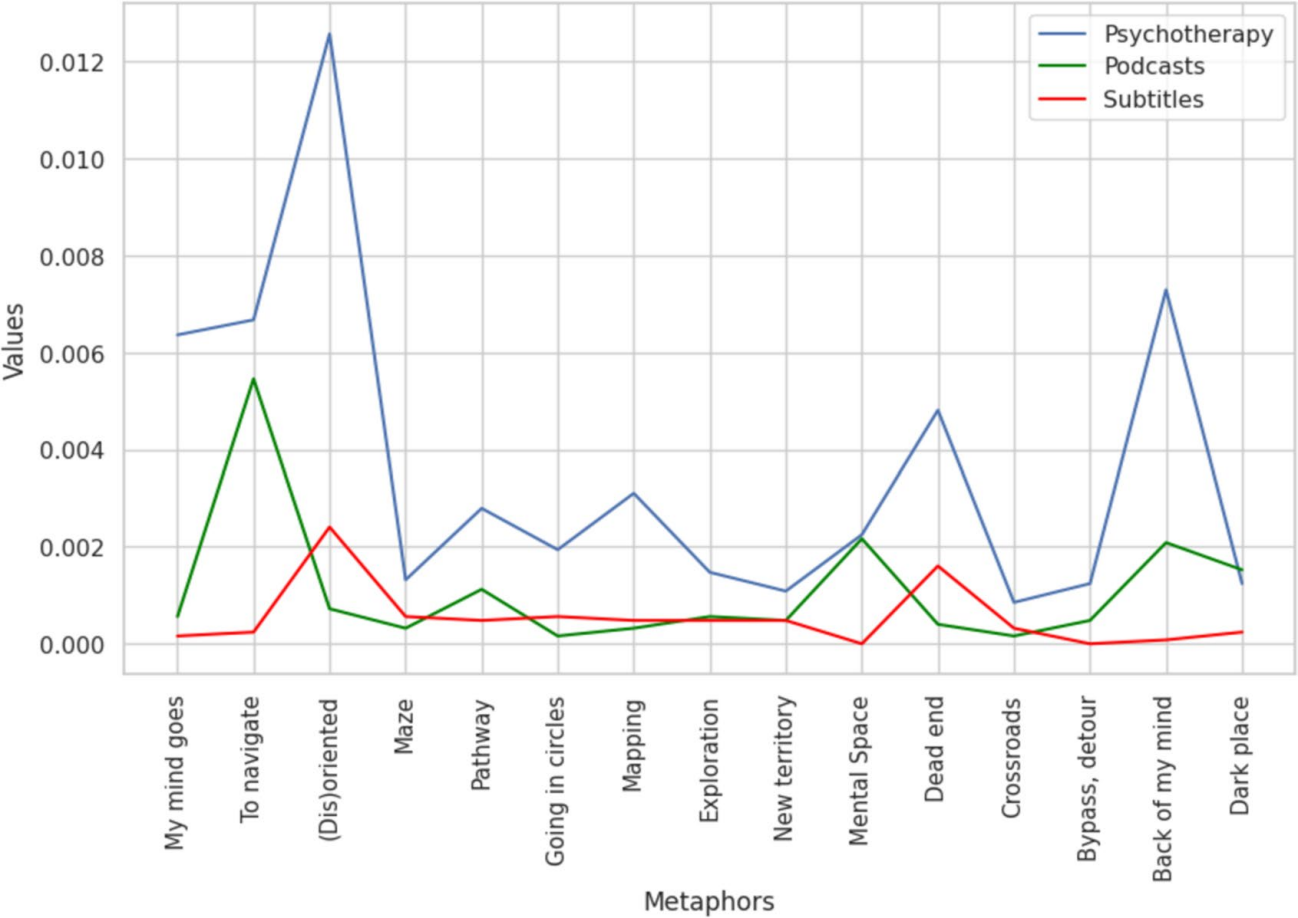
Frequency of occurrence of spatial/navigational metaphors irrespective of the context

In contrast to the previous dataset, this one included all instances of metaphoric use of navigational and spatial words, regardless of the context. However, this broader inclusion did not impact the differences between the datasets (*H* = 20.223, *p* = < .001). Notably, the use of spatial metaphors remained considerably higher in psychotherapy compared to podcasts (*U* = 192.0, *p* = .001, *d* = 0.549), as well as in comparison to movie subtitles (*U = 211*, *p* < .001, *d* = 0.792). Similar to the findings in the previous analysis, no significant difference was observed in spatial language use between podcasts and movies (*U* = 151, *p* = 0.112, *d* = 0.279) (shown in Fig. 3). Again, this effect was not driven only by a few concepts. When we omitted the top three spatial concepts from the analysis, they were still more prevalent (*H* = 16.264, *p* = < .001) in psychotherapy transcripts than in podcasts (*U* = 122.0, *p* = .008, *d* = 0.623) and movies (*U* = 138, *p* < .001, *d* = 0.867). These consistent results further support our hypothesis that spatial/navigational metaphors are more frequently employed during psychotherapy in comparison to more regular conversations. Analyzing these datasets, we again used the additional “Impartial categorization” dataset. Identically, the results were not affected (*H* = 14.150, *p* = < .001). The usage of metaphors significantly differed between psychotherapy and podcasts (*U* = 90.0, *p* = .005, *d* = 0.556), as well as between psychotherapy and subtitles (*U* = 95.0, *p* = .002, *d* = 0.939).

#### Non-spatial concepts

**Fig. 4:**
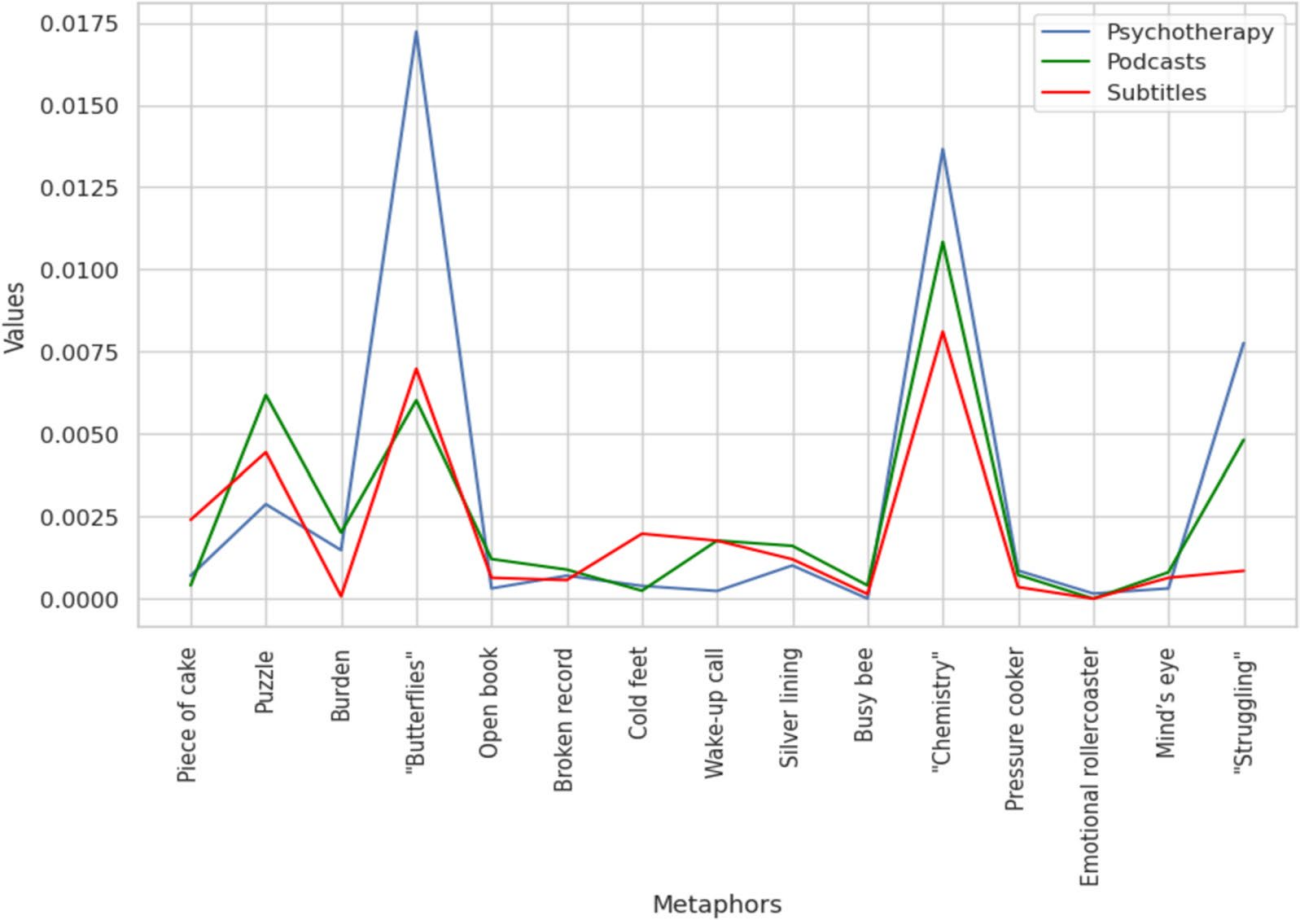
Frequency of occurrence of non-spatial/navigational metaphors across datasets

To ascertain whether the prevalence lies solely in spatial/navigational metaphors or if psychotherapy exhibits prevalence with all possible metaphors, we conducted a similar analysis focusing on common non-spatial metaphors. As predicted, this analysis revealed no significant differences between the datasets (*H* = 0.682, *p* = 0.710). To ascertain that we chose concepts that are indeed not associated with space, we conducted an additional analysis on the “Impartial categorization” dataset that consisted of non-spatial concepts identified by at least 80% of respondents unfamiliar with the article. This analysis yielded similar results and did not show significant differences (*H* = 0.306, *p* = 0.857) (shown in Fig. 4).

The absence of differences in employing non-spatial metaphors between psychotherapy and other conversational corpora underlines a crucial finding. It indicates that the extensive usage of spatial and navigational metaphors might be a distinctive characteristic unique to psychotherapy or self-analysis, highlighting the phenomenon of mental navigation.

## Qualitative analysis

The primary objective of the qualitative analysis (see Qualitative methods for methodology) was to investigate and identify the manner in which therapists and clients employ spatial language to describe their introspective experiences. In addition, we aimed to discern patterns and observe dynamics associated with mental navigation throughout the psychotherapeutic process. Thorough data collection facilitated the identification of the most prominent concepts emblematic of mental navigation, with illustrative examples provided for each within the Supplementary table. Nevertheless, it is crucial to acknowledge that the database contains a multitude of additional instances that further underscore the richness and diversity of spatial metaphors present within the database.

To further illuminate the role of mental navigation in the psychotherapeutic process, we present contextual examples that showcase its utilization. One prominent way of representing the mind’s spatial dimension is compartmentalization, a concept long-standing in the aforementioned idea of “Memory’s theater.” In the following example [159], the therapist (T) engages in a conversation with the client (C) about the importance of utilizing one’s mental capacity to its fullest potential. The therapist introduces an analogy comparing the mind to a house where certain compartments have been locked and are inaccessible. The therapist suggests that by unlocking these compartments and accessing all parts of the mind, the client can enhance various aspects of their life, including work, emotions, and overall well-being. The dialogue highlights the significance of mental exploration and the potential benefits of mental navigation in expanding one’s cognitive capacity for more comprehensive problem-solving and self-understanding:

T: That's what I'm saying. The analogy breaks down, but even with a desk job, you may do less walking, but you still have to walk. And you, who love to use your mind, to *compartmentalize and cut off pieces of your mind that just is a waste of mind space, compartments that you can otherwise be accessing.*

C: For what, access for what?

T: *For your work, for your life, for your emotions, for everything. If you're living in a house, locked half the doors, and decided you can't go in them, you only have half the house to work with!*

In the context of our previous discussions, therapy can be likened to a process of finding direction and orientation, with the therapist guiding the client through their inner world. The following conversation [160] exemplifies this idea. The client compares their mental state to being trapped in a maze, feeling disoriented and stifled by the overwhelming complexity. The therapist acknowledges the disorienting nature of a maze and recognizes the client’s desire for guidance. The therapist introduces the concept of a compass as a tool for orientation, initially surprising the client. However, the therapist explains that a compass is rather a tool for facilitating navigation than an intricate and restricting space like a maze or an ocean.

This example demonstrates how the client’s perception of their inner world can be shifted by using different words to describe it. Instead of feeling trapped in a maze or an ocean, the client is encouraged to see therapy as a compass, offering a way to navigate and find their path instead of passively being lost. Overall, this dialogue highlights the power of language and metaphor in shaping the client’s mental navigation:

C: […] I'm conscious of that, as well*. But it's funny when you said that he provides orientation, the picture in my head was me in a maze; (laughs) only knowing how to get from here to there, not how I'm going to get out. It's funny because I felt both comforted and oriented and disoriented. It's really strange because I'm oriented for the time being, but overall I'm lost. The option is to be in a sea, an ocean, or to be in a maze. (laughs) Are there no other options? What other orientations are there? If I say to you "orientation", what picture do you see in your head?*

T: *A compass.*

C: Okay, so *just directions?*

T: Right. You're associating a structure or a lack of structure. *I'm associating essentially a type of map. You're associating to a form of something or a lack of form. I'm associating to a tool orient, which is a compass. A compass is a tool. You're not in a compass like you're in a maze or in an ocean. You're just using it as a tool.*

C: *I would never think of a compass.* (chuckles)

T: *So maybe, to stretch the metaphor, maybe therapy is the compass.*

C: Okay.(laughs)

T: *The tool to orient you.*

In contrast to the previous example, where a maze symbolized complexity and lostness, the next instance [161] sheds light on a possible positive aspect of the inner maze – our ability to navigate it using emotional cues. This realization prompts the client to conceptualize emotions as valuable guides for decision-making. Employing the metaphor of a maze, the client describes negative emotions as walls that restrict movement in directions they perceive as unproductive. This exchange underscores the significance of emotional navigation as it empowers clients to make informed and more meaningful choices. This idea, perhaps, can also be used by therapists to guide clients in recognizing areas they should avoid, such as “jumping to conclusions,” where negative emotions are often triggered. However, in some instances, on the contrary, it may be necessary to explore and restructure negative aspects, as anecdotally depicted in phrases like, “If you run away from problems, they will catch you at the most inopportune moment.” Thus, attending to emotional cues during navigation can serve as an indicator of both places to avoid and places to dig deeper:

C: And that was something that, for him, seems to have worked out. I was thinking that it wouldn't you said, *using the emotions as a way to guide yourself, I really see it as a kind of a maze in which the negative emotions sort of form the walls of the maze and prevent you from going in a certain direction because you feel that it's not helpful to you to go in that direction so you go in another direction.*

When a physical map presents uncertainty with unmapped places, it might evoke fear due to possible threats present in an unexplored territory [162]. The same seemingly applies to the cognitive “territory” as the database revealed a sufficient use of the “unexplored territory” concept. The following is a specific example of such an instance [152]. The client explores their emotions concerning their marriage and uncertainty about the future. Using the metaphor of a road, the therapist portrays the client standing at a point of choice, unsure of which path to take. Delving deeper into the metaphor, the therapist highlights one road leading to a destination without fulfillment or happiness while the other represents the client’s wife. However, the latter road feels like uncharted territory, invoking fear and apprehension. The therapist’s proposal encourages active engagement in the investigation of this uncharted territory and urges the client to map out the unknown aspects:

T: You really feel *you're at a choice point* and I guess you kind of felt it a while, but maybe it feels more narrowing now. *You don't know which road to take. It's like you don't know if the road with your wife leads anywhere.* It never leads it to a nice place that's really happy to live.

C: Yeah. It's like when you hear...when I talk with friends and they're talking about the future with their wives or...well, okay, we'll say with their wives and their families and I never really even feel a sense of the future for us.

[…]

T: You don't know why you have the feeling. Kind of the effects of having a feeling is kind of like *you can't feel like that road really...you feel like that road doesn't...it doesn't go anyplace.* You can't feel it. You don't feel *that it goes anyplace, but the other road* may be your wife*. God, that's really unknown territory. It's scary territory. It's not mapped. You have to create your own map and find your way. That's pretty scary.*

The following example [163] is truly captivating due to the therapist’s skillful investigation and effective communication with the client. The therapist acknowledges the client’s difficulties and identifies them as practical emotional challenges. By reframing the issue, they assist the client in understanding their feelings of being stuck, passive, and uncertain. The therapist demonstrates how adopting a new perspective can help the client overcome anxiety and negative emotions. Throughout the conversation, the central theme is the exploration, learning, and recognition of problematic areas causing negative experiences. Essentially, the therapist encourages the client to engage in mental navigation, uncovering and becoming aware of the aspects that govern their actions but remain unnoticed:

T: Your experience is, in a way, of being faced with a practical problem. Namely, what do you do about this? Both, in a way, while you're here and more broadly. (inaudible) *come if it feels like . . . we're not getting you through this impasse?* […] *And I think this impasse figures large in your thinking about this and how you're doing, in general. Is that right?*

C: Yeah.

T: Yeah. So, that's a practical problem. And it's an important one.

I think, in a surprising turn of events, I think that that practical problem, which is a real one and one that deserves serious consideration. Also conceals an emotional issue or you could say problem. *Which is that what the impasse feels like, what the stuckness and passivity and uncertainty* all feel like . . . particularly . . .(pause)

[Skipped replica]

T: I don't think my laying it out this way is any kind of solution or procedural manual . . . for how to deal with these situations. I'm saying *I'm trying to reframe a little bit where I think you get stuck and why.* And how I think you-and I think the kind of things that we can work on to help make it better. Which involve . . . gradually coming to tolerate and . . . deal with the anxiety of and get more clarity about or understanding of what's going on when this happens. Why it's so overwhelming. *Getting used to that in a way that helps you feel more settled when it's going on, so that you can think, in a sense, where you're actually at in any individual situation where you're stuck and this comes up.*

C: Right.

T: […] *And this is often the way that psychoanalysts (inaudible) analysis work is that you wind up stuck in the same place in the treatment with the analyst that you are everywhere else. And you don't find an answer or a solution or a strategy so much as you come to get used to gradually think your way through, become less anxious and churning and paralyzed by the feelings that come up in the situation or around it, so that you can think and operate and get a sense of how you feel and where you're coming from when the problem comes up, in a way that you couldn't before*

Note that numerous additional contextual examples were collected and analyzed. However, here, we presented only those that we consider the most vivid and easiest to grasp.

## Discussion

In general, psychotherapy has proven to be effective in the treatment of a wide range of mental conditions [9–15], yet it still lacks well-defined cognitive and neural explanatory mechanisms (e.g., [20, 164]). In our research, we aimed to demonstrate that the phenomenon of mental navigation has the potential to become a general framework that helps to understand the process of psychotherapy.

Drawing upon recent research, a link between the neural mechanisms of mental navigation and changes on the conceptual and phenomenological levels has been proposed [23]. This intriguing connection prompted us to hypothesize that mental navigation may have a significant role to play in psychotherapy, as it is well-known that patients delve deeply into their cognitive space, exploring the ways they think and feel, aiming to optimize these processes and reduce stress. Further, spatial language commonly used in psychotherapy caught our attention as a robust indicator of these underlying neural and cognitive mechanisms, along with the specific phenomenology that resembles navigating an intricate physical space.

Through quantitative analysis, we observed a tendency for these spatial and navigational metaphors to be more frequent during introspective experiences in psychotherapy. However, its occurrence appeared less prevalent in regular or fictional conversations, which could suggest a potential significance of spatial language in describing one’s phenomenological experiences. Additionally, our qualitative analysis provided practical insights into how clients and therapists utilize spatial language to navigate their cognitive landscape, shaping their search for understanding and bringing clarity to their inner situation. By drawing parallels between mental navigation and psychotherapy, we showcased the existence and potential influence of mental navigation in the therapeutic process, making it a promising new avenue for understanding psychotherapy and the transformative changes it brings about.

### Mental navigation as an explanatory framework

As outlined in the introduction, a variety of widely recognized psychotherapy frameworks often share a common thread: the need for self-analysis. This process involves exploring our cognitive space, recognizing preexisting patterns, and consequently restructuring or changing them. Notably, these frameworks have different principles, techniques, and requirements for clients. Yet, what lies at the core of a majority of the approaches is close self-examination. No wonder that this ability was of interest to ancient philosophers [165] and has been thoroughly explored in the history of psychology [166–168]. After years, contemporary mental health research highlights the value of heightened metacognitive awareness – the ability to know our thoughts and feelings – as it links to better mental health outcomes [169–174], underscoring the significance of our ability to introspect for achieving therapeutic results.

However, despite its importance and widespread application, to our knowledge, no comprehensive theory exists outlining the components and mechanisms of this introspective ability. Here, we built upon recent research that introduces a connection between higher-order cognition and the functions of place and grid cells [21, 22]. These studies suggest that these cells encode our abstract knowledge and potentially form conceptual (cognitive) maps, similar to how spatial information is organized. Moreover, studies on conceptual spaces [58, 59] and mental metaphors like mental number lines or the spatial organization of temporal information [62, 69–71] provide evidence for the fundamental role of space in our cognition. Based on these frameworks, we suggest that the process of psychotherapy can be conceptualized as mentally navigating [23] these conceptual spaces with the aim of recognizing and revising problematic areas.

To summarize the idea, according to our framework, individuals often seek therapy due to the complexities and challenges within their conceptual maps, encompassing both cognitive and emotion-related issues. On that basis, we consider the crux of effective psychotherapy, whether cognitive, dynamic, or any other, to be centered around a unifying requirement: the need to expand, update, restore, or restructure one’s conceptual map. Mental navigation is a process that can facilitate these changes through careful identification, exploration, and manipulation of problematic places within one’s cognitive space. With this unifying ability, mental navigation surpasses specific psychotherapeutic modalities and encompasses any approach that involves exploration and the enhancement of self-understanding, thus potentially playing the role of a general explanatory framework for psychotherapy.

In this context, conceptualizing the cognitive landscape of a client as a map offers an intuitive way of understanding the therapeutic process. Just as there may be issues with a physical map, clients’ conceptual maps may also require exploration and updating, which therapists facilitate during psychotherapy. This perspective is echoed by a therapist whose session transcripts were included in our analyses. They metaphorically describe the essence of psychotherapy to their client using the following lines: “Therapy is a compass… The tool to orient you” [159] or “In a nutshell, it’s basically why people come to therapy because they feel stuck” [175].

Thereby, the therapist’s role is akin to that of a skilled navigator, proficient in traversing unexplored territories within the client’s psychological space. This expertise involves a deep understanding of how to navigate these psychological landscapes effectively, restructuring problematic areas as necessary, and, most importantly, guiding the client through this transformative journey. As previously mentioned, various therapeutic approaches can facilitate this process. Cognitive techniques encourage clients to map new or alternative thoughts, while dynamic psychotherapy techniques suggest delving deeper, forging connections, and interpreting previously hidden ideas and memories. These techniques can differ significantly from one modality to another and encompass different non-introspective requirements, such as learning new skills and behavioral reactions. Yet, we suggest that mental navigation serves as a backbone and a starting point for a majority of techniques aiming at shifts on conceptual and phenomenological levels.

### Leveraging mental navigation in psychotherapy

In light of the preceding discussion and the arguments presented, a pertinent question arises: Can the concept of mental navigation, in conjunction with spatial language, be explicitly harnessed in psychotherapy to yield more profound and meaningful outcomes? To address this question, it is important to clarify that mental navigation is a multifaceted concept encompassing neural, cognitive, experiential, and potentially other dimensions. Hence, mere usage of spatial language does not automatically equate to mental navigation. Instead, mental navigation is fundamentally about the process of actively exploring and revisiting one’s cognitive space, whether or not it is articulated using spatial metaphors. However, what is critical is that spatial language might serve as a tool for expressing, describing, or sharing these internal relations or mental maps with a therapist who does not have direct access to them. On the other hand, therapists might use spatial language as a tool for triggering or prompting mental navigation while also influencing its direction and focus. To use an analogy, spatial language is a rudder to control the mental navigation machine – when the position of a rudder is changed, the direction of the machine is also changed. On that basis, we suggest that therapists can leverage spatial language intentionally and explicitly to facilitate mental navigation.

Previous studies have shown that it is unlikely that metaphors alone can facilitate meaningful changes in short and long time periods review, (for a recent review, see [121]). Thus, we warn against simplistic interpretations for increased usage of spatial metaphors as the mechanism of therapeutic transformation. However, as there is more consensus that metaphors can facilitate the therapeutic process itself [121], we suggest that skillful and well-timed usage of spatial (and probably other) metaphors can facilitate the exploration of a client’s mind. This exploration, in turn, might indeed lead people to beneficial therapeutic outcomes. Based on that, it is pertinent to study how to facilitate this process to help the client successfully explore their conceptual space, find problematic places, and try to restructure them.

Equipped with the understanding of mental navigation mechanisms and awareness of the ubiquity of space in the way we organize our mental representations, therapists can probably facilitate the process for both themselves and their clients. First of all, as it is often difficult for a client to express previously undefined feelings and understand their inner world, therapists can enhance clarity for clients by explicitly conceptualizing their inner state in spatial terms. Insofar as spatial terms are familiar to everyone and applicable in various contexts, this approach might enable clients to grasp their introspective experiences more vividly, facilitating a deeper understanding of their feelings and thoughts. To reiterate, the critical component of this approach is the ability to use spatial metaphors with the understanding of how and why they can work successfully instead of using them haphazardly without a clear goal. Furthermore, therapists skilled in mental navigation could help refine the clients’ introspective process by tracking their cognitive journey. It means that therapists can identify the paths taken and directions avoided, which might allow them to better orient in the psychotherapeutic process by constructing, in a metaphorical sense, a comprehensive cartography of the client’s psychological space.

Additionally, by paying close attention to the client’s metaphorical descriptions of the introspective experiences, therapists can gain insights into relevant cues or triggers and how they relate to the client’s problems. For example, if the client “does not want to go there” or “there is a dark place,” it might prompt the therapist to explore and map the problematic place so that it is no longer intimidating. Another example would be “stuckness” or a “dead end,” which, in the same fashion, may give the therapist a clue for investigating what exactly constitutes the “dead end” phenomenology as well as identifying alternative paths. Besides, as briefly noted in the introduction, another fruitful way of employing spatial language is by viewing it as a proxy for detecting progress, steps, or intricacies that signify a client’s advancements or lack thereof. Although therapists probably already employ these methods quite naturally and intuitively, by adopting the metaphors into their practice more strategically, they can convey abstract ideas more vividly or, on the contrary, neutrally, thus influencing the dynamics of the process. Potentially, there are many more diverse applications of spatial language for enriching therapeutic processes, and we leave the opportunity to develop them for future studies and psychotherapy practitioners.

A final noteworthy remark within this subsection concerns the relative scarcity of spatial language in everyday and fictional conversations, which raises the question of why such language is less prevalent in these contexts. One possible explanation could be that, in contrast to psychotherapy, casual discussions and artistic dialogues often lack the requirement for a close self-examination. As a result, these contexts may not evoke the mental navigation process and, subsequently, the utilization of spatial metaphors that are particularly well-suited for its articulation.

### Limitations and future research

There are several important issues that fall outside the scope of this paper and should be addressed in future research. Firstly, while we used podcasts as a more casual form of conversation, it is possible that a certain percentage of them are still scripted or that the participants are at least prepared for the discussion. Secondly, although a qualitative analysis of podcast and movie transcripts was beyond the scope of this paper, it is possible that some alternative uses of spatial language could have emerged from those contexts, which would insinuate that variations of mental navigation also appear in everyday conversations. These questions will hopefully be addressed in future research. Thirdly, while the manual categorization approach helped us to pick the most prominent examples, it does not guarantee an exhaustive list of metaphors. This could be improved in future studies with a more comprehensive analysis, including the incorporation of automated techniques and larger text corpora. Fourthly, the use of spatial language in each psychotherapy session might depend on various factors, such as the therapist’s style [109, 110], the client’s preferences, and cultural background. It is also crucial to recognize that this research solely focused on the English language corpora, promoting future studies to broader language inclusion as individuals might apply spatial and navigational metaphors differently depending on language specificity. Lastly, while the database of psychotherapy transcripts encompasses a majority of well-established psychotherapeutic modalities (see Qualitative methods), it does not include such approaches as, e.g., EMDR, psychodrama, exposure or play therapies, and thus we cannot conclude that all therapies rely on mental navigation.

An additional limitation of our analysis concerns the sessional (or episodic, in the case of podcasts and movies) nature of the transcripts. It is possible that some clients (or podcast guests or movie characters) were prone to use metaphors much more than others, which could affect the results. Due to the research being conducted manually on large text corpora, performing an analysis addressing this issue was not conceivable at the time. Thus, we encourage future research to conduct more thorough analyses that would allow us to identify patterns of spatial metaphor use across different individuals or conditions, as well as quantify the potential occurrences of metaphors per given text segment.

Furthermore, a potential limitation lies in the imperfect comparability of the podcasts dataset to the two other datasets, as it represents conversations from a small time period (2019–2020) compared to longitudinal data in psychotherapy and subtitles databases (1970–2015). In addition, we did not conduct a close analysis and categorization of metaphors into novel metaphors, conventional metaphors, metonymy, allegory, anecdotes, or idioms. Instead, we concentrated on the fact of using space in its figurative meaning more broadly. However, future studies should concentrate on these differences and explore whether there are any significant discrepancies in their usage in therapy. Moreover, though we addressed the issue of ambiguity by creating two datasets with different counting methods (“Introspective spatial metaphors” and “All spatial metaphors”), there still may be a possibility that our analysis was not robust in the face of ambiguous cases of metaphor use beyond just connotation in which they were used. Hopefully, future research will tackle the mentioned issues.

It is also important to note that within the proposed framework, distinct types of navigation can be studied. This paper does not specifically delve into the realm of imagined spatial navigation, a subtype of mental navigation. Other types, such as virtual reality navigation (as experienced in computer or VR gadget games), fictional navigation (guiding through a story in a novel or a movie), and “high-level” navigation (pertaining to scientific or spiritual realms), are also recognized but not the central focus of this study. The concentration here revolves around what can be termed as epistemic navigation. In the context of mental navigation, epistemic navigation pertains to the exploration and revision of established knowledge within the mind. However, we hope that this paper will prompt studies involving a broader range of mental navigation types.

Overall, despite analyzing the patterns and prevalence of spatial language in psychotherapy as well as suggesting explicit use of it, our research does not definitively demonstrate the effectiveness of mental navigation in therapeutic outcomes. Given the limited data at our disposal, we were unable to draw any inferences about the outcome of therapeutic methods that use spatial language compared to non-spatial language. In the future, using datasets that are labeled with information about therapeutic progress or include subjective assessments of efficacy by the client or therapist would be useful. Furthermore, the database of psychotherapy transcripts contained a vast array of sessions with different clients and therapeutic approaches but offered limited instances of prolonged therapy sessions for in-depth analysis. In light of these limitations, our focus was directed toward providing a more extensive and theoretical exploration of mental navigation as a fruitful new direction for future research.

Therefore, future studies could focus on investigating whether explicitly leveraging mental navigation during psychotherapy sessions leads to improved therapeutic results. To address this issue, we propose conducting a study with a randomized controlled design, incorporating three distinct conditions: therapists who do not use any metaphors, therapists who use spatial language to facilitate mental navigation, and therapists who use non-spatial metaphoric language for comparison. Moreover, we encourage psychotherapy practitioners to take the concept of mental navigation, as outlined in this paper, as a basis for developing and testing new therapeutic techniques. However, it is also crucial to urge the practitioners to approach these new techniques with scientific rigor and ethical considerations.

## Conclusion

This paper presents a comprehensive exploration of mental navigation as a novel framework to comprehend the intricacies of the psychotherapeutic process and the subsequent transformations. Drawing upon the history of spatial navigation research, we analyzed focused on several prominent studies on the cognitive and neural mechanisms underpinning mental navigation – our ability to navigate conceptual spaces – postulating its fundamental role in self-analysis. In addition, we hypothesized that spatial language and navigational metaphors serve as indicators of mental navigation during psychotherapy. To test this hypothesis, we employed both quantitative and qualitative methods.

The results of the quantitative analysis showed a noteworthy difference in the frequency of spatial language use during psychotherapy compared to more casual conversations and fictional conversations. This discrepancy potentially suggests that spatial and navigational metaphoric language hold particular prominence in psychotherapy, highlighting their relevance in describing introspective experiences. Furthermore, the qualitative analysis of psychotherapy transcripts helped us to show how clients and therapists utilize these metaphors to navigate their internal cognitive landscapes. These examples shed light on the dynamics of this process, elucidating how mental navigation is instrumental in exploring, revising, and restructuring cognitive patterns during psychotherapy.

With these findings, we seek to pave the way for future research and encourage psychotherapy practitioners to embrace the concept of mental navigation in their therapeutic practices. By integrating this framework, they can explore innovative interventions and approaches to enhance the therapeutic process and improve mental health outcomes for their clients. The incorporation of mental navigation principles in psychotherapy has the potential to advance the field and foster positive and transformative therapeutic experiences.

### Supplementary Information

**Additional file 1. **[177–217].

## Data Availability

The data in this study was obtained from distinct third parties. The Psychotherapy Transcripts Database by Alexander Street Publisher [151] is publicly available for non-commercial use and can be accessed through institutional (trial-period) subscription upon request from the publisher via this link: https://search.alexanderstreet.com/counseling-therapy/browse/title?showall=true&f[0]=document_type_facet:Session%20transcript. The Spotify Podcasts Dataset [155, 156] is also publicly available for non-commercial use and access can be requested personally through the following link: https://podcastsdataset.byspotify.com/. Open-access movie subtitle corpora are freely available for both research and commercial usage and are downloadable from https://www.opensubtitles.org/en/search/subs. To download files with raw data and codes produced by us, please refer to this link: https://github.com/mykytakabrel/spatial-metaphors/tree/main. Further inquiries can be directed to the corresponding author.

## Abbreviations

CBT: Cognitive-behavioral therapy
JTC: Jumping to conclusions
REBT: Rational emotive behavior therapy
EFT: Emotion-focused therapy
BPD: Bipolar disorder
OCD: Obsessive-compulsive disorder
ADHD: Attention deficit hyperactivity disorder
PTSD: Posttraumatic stress disorder
CTRL: “Control” button
EMDR: Eye movement desensitization and reprocessing
MIP: Metaphor Identification Procedure
