# Supplementary material for: The journey within: mental navigation as a novel framework for understanding psychotherapeutic transformation
Source: BMC Psychiatry. 2024 Feb 1;24:91. doi: 10.1186/s12888-024-05522-8 (PMC10835954; doi:10.1186/s12888-024-05522-8)
Supplement: Supplementary file 1 — Additional file 1. [177–217]. [file 12888_2024_5522_MOESM1_ESM.docx]

**Supplementary table.**

Usage of spatial/navigational metaphors

| **Concepts** | **Clients** | **Therapists** |
| --- | --- | --- |
| **Walking mind** | I think *the way my mind goes* first is towards a negative side of things as opposed to looking on the brighter side [163]. | See *if you can reflect on and see where your mind goes,* and see if any, you know, flash comes up or memory comes up [164]. |
| **Going somewhere** | We *go there and it is like, hmmm, yeah. It is like the brain shuts down.* It is like *I don't want to go there.* I don't want to think about it [165]. | *Why? Why don't you go there here but you go in other places?* [166] |
| **Steps** | *So far, this is a small step in that direction. Hopefully, there will be other steps along the way that take me there* [163]. |  |
| **Getting lost** | *I definitely feel needy or like my need for direction is so intense. I really, absolutely feel lost without it* [164]. | From what you're describing, *you really feel lost* [167]. |
| **Being stuck** | *I guess when I feel sad I feel like I'm stuck and I'm not going anywhere* [168]**.** | *I mean, in a nutshell, it's basically why people come to therapy because they feel stuck* [169]. |
| **Jumping** | I will often immediately *jump to the conclusion* that they are talking about me and I've done something awful [170]. | **…** but you seemed to *very quickly jump to that conclusion and then extrapolate the drama before you had any evidence whatsoever* [171]. |
| **To go in circles** | See, you know, *it makes me go in circles then though* [172]. |  |
| **Obstacles** | *Yea. I mean, at least for me. Maybe only because I built it up to be this huge obstacle in my head* [173]. | I wonder if there's any chance *there are obstacles inside to* make you more money, somehow *there are emotional ones?* [174] |
| **From point A to point B** | *Well, this is just how therapy goes, you know? Sometimes you don't get directly from point A to point B because that's not the obvious path or something we've been walking* [175]. | I think you're interested what is this, to the degree that it can help you understand and put into perspective even some timeframe stuff around it and *how you're going to get from point A to point B* [176]**.** |
| **Dead end** | Well, you know, I guess I feel like *I've kind of come to some dead ends*, you know, talking about things, you know, and, you know, kind of in general [177]. | And that's probably part of what's pulled you together is sort of being able to feel attached like that*, and so the immediate impasse can be sort of circumvented* [178]. |
| **Crossroads** | *And, I don't know, I mean, I feel as if we're at kind of a junction, a crossroads. And I'm kind of interested in exploring that a little bit* [179]. | We've learned certain things and have come so far, but now *it feels to both of us like we're turning a corner in some way. We're at a fork in the road, or something like that* [180]. |
| **Bypass, detour** | Okay. I mean, *I don't mind the detour.* I just, *when we're detouring I'm not steering the direction anymore and* so (pause) which I don't mind [181]. | You mean like you can see something important there but *you go around the bend and take a detour* [182]. |
| **Navigation** | But I think I'll have to, you know, feel these feelings for a while, which is hard. But I know that it's the best thing for me. But it's... Like just *navigating all the dynamics and the guilt coming from so many places* is-it's really hard [183] | I mean, I'm just thinking about how much struggling really, really affected you, it really affected you. And you were kind of my sense is that**you** *were trying to navigate that, and you were having to navigate that on your own* [184]. |
| **(Dis)orientation** | It doesn't feel very helpful *in this space*, but to me, I feel so confused and so terrified. And I keep thinking of this kind of analogy of if you fall into the pool and you want to get to the top but you open your eyes and *you're disoriented and you don't know which way is up* [185]. | When you're feeling down, it's kind of you get into a very *introspective self-examination thing and kind of makes you feel disoriented* and very unsure of yourself, kind of very shaky, not sure what kind of *ground you're standing on*? [186] |
| **Maze** | I was thinking that it wouldn't you said, using the emotions as a way to guide yourself, *I really see it as a kind of a maze in which the negative emotions sort of form the walls of the maze and prevent you from going in a certain direction because you feel that it's not helpful to you to go in that direction so you go in another direction* [187]. | And it's kind of as *a way of finding a way out that maze* so that you can trust your own judgment about other people and yourself [188]. |
| **Pathways** | Of course *the right road always seems the most difficult road to follow. (chuckling) You know the road...you know is right for you and you know the thing to do is just...it seems like it's...it's the most difficult thing to do* [189]. | Even if there were something, I guess *I just want you to walk down that pathway* [190]. |
| **Maps** | How do you create a mental map you know? I feel like my mental maps are written in invisible ink. I'm not sure what chemical reaction causes them to disappear or to reappear. I have maps of my creditors, there's maps of my professional obligations and relationships. Maps of my friends [191]. | *God, that's really unknown territory. It's scary territory. It's not mapped. You have to create your own map and find your way* [192]. |
| **Mental exploration** | *I feel that I'm exploring more and more of the terrain around me* [193. | Well then we'll talk about it now, the How-to calm yourself, *so that you get yourself to the place of doing the cognitive investigation* [194]. |
| **Journey** | But that sort of thing is not, it's a nice thing to think about to say, but it's different. It's a *long journey of really accepting change* [195]. | *I'm here to share with you a journey through a different way of thinking* [196]. |
| **Territory** | I don't really understand that. *I can't go into unexplored emotional territory, because the territory that you're presenting to me is the territory that I know*, because you present yourself to me the way I think I present myself to you to some degree [197]. | We've talked about a couple of things about that, *which are it's very unfamiliar territory to you. I mean you don't know your way around it, at all* [188]. |
| **Places** | *There's like, it's like a really deep, dark thing way down there somewhere.* There's this...I don't know, it's all I can say. It's like a key to myself that, you know… [189] | You guys have your triggers, as most couples and people do. I see when you guys get triggered you go to *a sort of dark place* quickly [198]. |
| **Location** | Like I can't I can't articulate or even understand the things I've gone through. *Like I can't locate myself.* So yeah [199]. | *You're sort of putting pressure on me to locate you amidst ambiguity and confusion, yeah?* [200] |
| **Guidance** | And I trust that *you know how to guide me into my psyche* to figure more of this stuff out [197]. |  |
| **Space** | I feel like for me the goal is not so much *to put myself in a mental space where* I think that everything that has happened to me had to happen, as it is *to put myself in a space where* I think that it's okay that things don't make sense, if that makes sense [201]. | Also, if you're thinking about particulars of *what might be in your mind in that space*. It's not just self criticism. Self criticism sort of sounds like it's like a shallow level of describing it [202]. |
| **Back of the mind** | You know? That's the speculation about maybe what I was - *what process was going on in the back of my mind*, you know? [203] | Now before I stop I want... I've... *in the back of my mind, I've been going around* trying to stick to what I was saying before [204]. |
